# Structural Plasticity and Immune Evasion of SARS-CoV-2 Spike Variants

**DOI:** 10.3390/v14061255

**Published:** 2022-06-09

**Authors:** Dibya Ghimire, Yang Han, Maolin Lu

**Affiliations:** Department of Cellular and Molecular Biology, University of Texas Health Science Center, Tyler, TX 75708, USA; yang.han@uthct.edu

**Keywords:** SARS-CoV-2, variants of concern, Spike proteins, structures, conformations, immune evasion, antibody escape, transmissibility, infection

## Abstract

The global pandemic of COVID-19 caused by severe acute respiratory syndrome coronavirus 2 (SARS-CoV-2) has significantly affected every human life and overloaded the health care system worldwide. Limited therapeutic options combined with the consecutive waves of the infection and emergence of novel SARS-CoV-2 variants, especially variants of concern (VOCs), have prolonged the COVID-19 pandemic and challenged its control. The Spike (S) protein on the surface of SARS-CoV-2 is the primary target exposed to the host and essential for virus entry into cells. The parental (Wuhan-Hu-1 or USA/WA1 strain) S protein is the virus-specific component of currently implemented vaccines. However, S is most prone to mutations, potentially shifting the dynamics of virus-host interactions by affecting S conformational/structural profiles. Scientists have rapidly resolved atomic structures of S VOCs and elucidated molecular details of these mutations, which can inform the design of S-directed novel therapeutics and broadly protective vaccines. Here, we discuss recent findings on S-associated virus transmissibility and immune evasion of SARS-CoV-2 VOCs and experimental approaches used to profile these properties. We summarize the structural studies that document the structural flexibility/plasticity of S VOCs and the potential roles of accumulated mutations on S structures and functions. We focus on the molecular interpretation of structures of the S variants and its insights into the molecular mechanism underlying antibody evasion and host cell-receptor binding.

## 1. Introduction

### 1.1. The Rise of SARS-CoV-2

Coronaviruses (CoVs) are enveloped positive-sense single-stranded RNA viruses which belong to the Coronaviridae family [1,2]. Before SARS-CoV-2, six human CoVs were known, namely HCoV-229E, HCoV-NL63, HCoV-HKU1, HCoV-OC43, Middle East respiratory syndrome coronavirus (MERS-CoV), and severe acute respiratory syndrome coronavirus (SARS-CoV) [3,4,5,6,7,8,9,10,11,12,13,14]. They all infect the upper and lower respiratory tract [3,4,5,6,7,8,9,10,11,12,13,14]. The most recent outbreaks were due to SARS-CoV in 2003 and MERS-CoV in 2012 [3]. At the end of 2019, a novel CoV was identified as the causative agent of a significant number of pneumonia cases in Wuhan, China. The World Health Organization (WHO) named this CoV as SARS-CoV-2 and the disease as Coronavirus Disease 2019 (COVID-19) in February 2020 [15]. Since then, SARS-CoV-2 has caused the devastating COVID-19 pandemic by rapidly spreading worldwide, and many variants have emerged. According to the WHO, by March 2022, over 476 million people had been infected, and over 6 million had lost their lives worldwide.

The genome size of SARS-CoV-2 is about 29.9 kb [16], and the genome encodes four structural proteins: Spike (S), envelope (E), membrane (M), and nucleocapsid (N) and 16 non-structural proteins (NSPs) [17,18]. The viral genome is protected by the nucleocapsid (N), further enclosed by the viral membrane and the membrane-associated structural proteins S, E, and M [17,18]. SARS-CoV-2 has several non-structural proteins, namely NSP1 to NSP10 and NSP12 to NSP16, encoded by genes located within the 5′-region of the viral RNA genome, which have various biological functions [19,20,21].

### 1.2. The Critical Player: The Spike Glycoprotein

**Figure 1:**
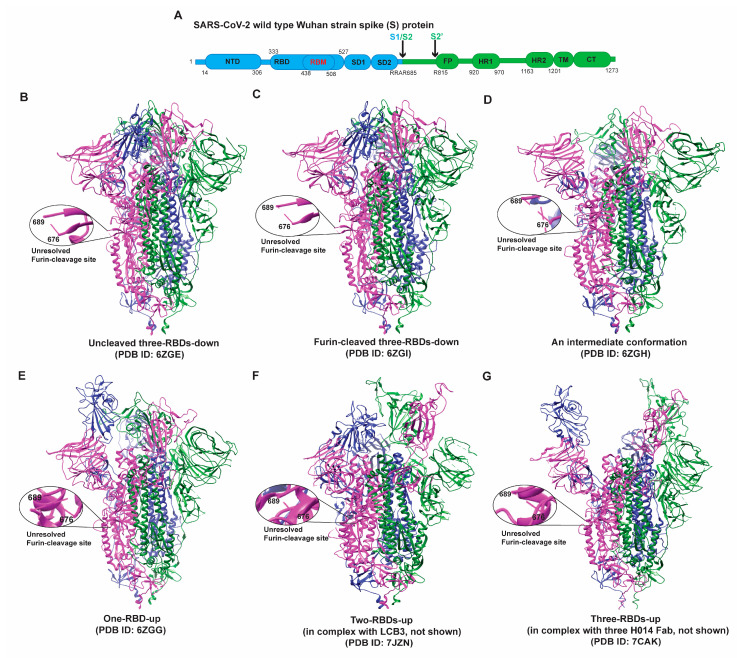
(**A**) Schematic map of the full-length (1273 amino acids) Wuhan-Hu-1 or USA/WA1 SARS-CoV-2 Spike protein. S1 and S2 subunits are represented in different colors. S1 subunit comprises the N-terminal domain (NTD), receptor-binding domain (RBD), receptor–binding motif (RBM) within RBD, subunit domain 1 (SD1), and subunit domain 2 (SD2), followed by S1/S2 cleavage site. S2 subunit contains S2′, a protease cleavage site, followed by fusion peptide (FP), heptad repeat 1 (HR1), heptad repeat 2 (HR2), transmembrane domain (TM), and cytoplasmic tail (CT). (**B**–**G**) Representation of S structures in different conformational states, including but not limited to an uncleaved, closed ((**B**), PDB ID 6ZGE), a furin-cleaved, closed ((**C**), PDB ID 6ZGI), an intermediate ((**D**), PDB ID 6ZGE), the one-RBD-up state ((**E**), PDB ID 6ZGG), the two-RBDs-up (in complex with LCB3) ((**F**), PDB id: 7JZN), and the three-RBDs-up (in complex with three H014 Fabs) ((**G**), PDB ID 7CAK). The furin cleavage site (682-RRAR-685) at S1/S2 was disordered and thus not resolved in the structures (**B**–**G**), as shown in the zoomed-in windows. There is no detectable structural difference (Figure S1) between un-cleaved closed (**B**) and furin-cleaved closed (**C**) conformations.

Like other coronaviruses, SARS-CoV-2 enters host cells with the help of Spike glycoprotein (S protein) [22]. The S protein, a heavily glycosylated type I membrane protein, is a trimer of heterodimers (S1/S2)_3_, in which S1 is the surface subunit and S2 is the membrane anchor subunit [23,24]. S trimers protrude from the viral surface with their large ectodomains. Each protomer contains 22 N-linked glycosylation sites [25]. S protein is initially produced as a precursor and later cleaved at the border of S1 and S2 (S1/S2 protease cleavage site) by a furin-like protease into two functional subunits: S1 and S2 subunits [23,24]. The S1 subunit containing an N-terminal domain (NTD) and receptor-binding domain (RBD) binds to the host receptor—the human angiotensin-converting enzyme 2 (hACE2) [26,27]. S2 subunit consists of fusion peptide (FP), heptad repeat 1 (HR1), central helix (CH), connector domain (CD), heptad repeat 2 (HR2), transmembrane domain (TM), and cytoplasmic tail (CT) as shown in the Figure 1A [17,27]. The fusion of the virus and the host cell membrane is the primary function of S2. Host proteases, mainly by the transmembrane serine protease 2 (TMPRSS2) at the cellular surface, are responsible for the further cleavage of S2 subunits, which may cause conformational changes in the S protein that facilitate virus-to-cell membrane fusion, a process of merging viral and cellular membranes [24]. In the endocytosis entry pathway, cathepsin L at the endosomal compartment cleaves S2 [28,29].

The S protein plays critical roles in transmission, viral pathogenesis, host immune responses, and evolution [23,24,30,31]. S binds the host cell receptor hACE2 to initiate the cellular entry and is hence critical for the transmission [23]. S is also involved in viral pathogenesis by activating the endoplasmic reticulum (ER) stress response [32]. Furthermore, it is one of the viral structural proteins exposed to the host immune system. Hence, it is a significant determinant of host immune response. As the primary target of antibody responses against SARS-CoV-2, S is both the target of antibody therapy and the SARS-CoV-2-specific component of both mRNA-based and adenovirus-based licensed vaccines [33,34,35,36,37,38]. S is prone to mutations, which lead to the emergence of SARS-CoV-2 variants with potentially altered pathogenesis and transmissibility, and antigenicity shift.

Many structures of the SARS-CoV-2 parental Wuhan stain (Wuhan-Hu-1 or USA/WA1) S protein have been determined using various structural tools. Employing cryo-electron microscopy (cryo-EM) or X-ray crystallography, the pre-fusion states of S ectodomain [23,27] and RBD-hACE2 complexes [26,39,40,41,42] have been determined. Similarly, pre-fusion and post-fusion conformations of detergent-solubilized full-length Spike proteins are also resolved by the cryo-EM [43,44]. Pre-fusion and post-fusion conformations of the intact S on the virion surface have also been studied using the cryo-electron tomography (cryo-ET) [45,46,47,48]. The resolved structures of Wuhan S have shown significant similarities to Spike proteins of other coronaviruses [49].

The domain organization and structures of a typical Spike protein in the pre-fusion states are depicted in Figure 1. Each S protomer can sample two primary conformations: “RBD-up” (“RBD” oriented up, hACE2-accessible) and “RBD-down” (RBD oriented down, hACE2-inaccessible) [26,27,43,46,50]. Consequently, the S trimer exhibits four major conformations: three/all-RBDs-down, one-RBD-up, two-RBDs-up, and three/all-RBDs-up (Figure 1). Un-cleaved and cleaved all-RBDs-down and an intermediate are hACE2-inaccessible states in which each S1 domain forms a V-shape structure with NTD at one side and CTD on another side, with RBD in the tip region in the homotrimer (Figure 1B–D) [37,38,39]. These structures make the NTDs lie in the periphery of the trimer and allow for establishing the interactions of all three NTDs with RBDs from the adjacent protomers. The S2 domains of three protomers form the helical bundle at the center of the trimeric structure. HR regions are then attached to the transmembrane domain and near the viral membrane. Of note, the furin cleavage site (682-RRAR-685) was not resolved in either the un-cleaved closed (Figure 1B, PDB ID 6ZGE) or the furin-cleaved, closed (Figure 1C, PDB ID 6ZGI) conformation. Interestingly, both structures (Figure 1B,C) exhibit very similar features at the atomic level without noticeable differences (structural alignment is shown in Figure S1).

One-RBD-up and two-RBDs-up (Figure 1E,F) [51,52] of the S protein have one or two RBDs open for binding hACE2 molecules. Three-RBDs-up conformation (Figure 1G) is generally observed when hACE2 molecules are present. The opening of RBDs causes various changes in the intra- and inter-protomer interactions within the trimeric S. The tracing of sequential changes in the trimer during the viral attachment has been attempted by the single-molecule fluorescence resonance energy transfer (smFRET) technique, which revealed a defined path for the conformational changes in the S protein that go through four different states [53]. The acquirement of D614G has increased the tendency of S to adopt receptor-binding competent conformations [54,55,56,57,58,59]. These early studies imply the potential changes in the structural ensemble and dynamics of S during virus evolution.

### 1.3. The Emergence of Different SARS-CoV-2 Spike Variants

SARS-CoV-2 viruses are under increased selection pressure from the vaccines, therapeutic approaches, and the host immune system. Whole-genome sequencing technology has allowed identifying the emergence of different SARS-CoV-2 variants. A list of variants and the mutations accumulated in their Spike proteins across different regions are summarized in the schematics and structures in this review. Alpha, Beta, Gamma, Epsilon, Delta, and Omicron were classified as the variants of concern (VOCs) [60,61,62]. These variants are more transmissible and possibly more pathogenic and immune–evasive. They carry accumulated mutations in the S protein. The resulting amino acid substitutions in S can impact the binding capacity to hACE2 and antibody recognition, therefore imposing constant challenges in current vaccine and therapeutic regimes. We will discuss the potential effects of conformational/structural plasticity of S during SARS-CoV-2 evolution in the context of virus transmissibility and immune evasion. This review explores the observations of immune evasion and altered virus transmissibility conferred by primarily studied S variants (Alpha, Beta, Gamma, Delta, and Omicron) and the mutation-induced structural dynamics of S variants. We also provide a schematic summary of approaches that have been widely used in the laboratory to characterize S variants. We then focus on the structural basis of S VOCs and their correlation with the molecular mechanism by which S VOCs evolve to escape antibody responses and meanwhile enhance virus transmissibility.

## 2. Virus Transmissibility and Immune Evasion of SARS-CoV-2 VOCs

### 2.1. Approaches Used for Characterizing Virus Transmissibility and Immune Evasion

**Figure 2:**
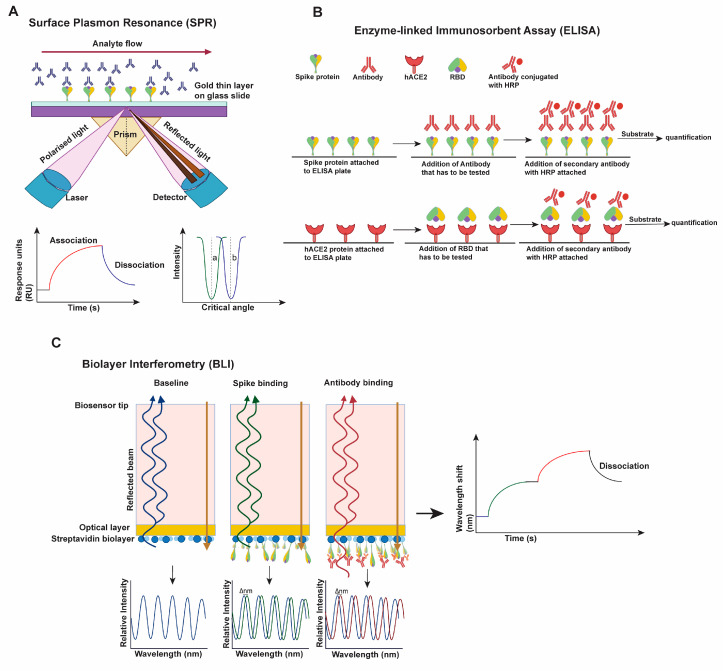
Schematics depicting the ligand-binding techniques used to characterize the binding of S to antibodies or hACE2. (**A**) Surface plasmon resonance (SPR) technique. SPR is an optical technique that can be used to analyze the binding of S protein with antibodies or hACE2. One of the interacting molecules is immobilized in the gold-layer sensor chip, and another interacting partner is flown through the microfluidic system. The interaction between the two molecules causes the change in the refractive index of the light, which is recorded as the response signal and measured in the response unit (RU). The plot generated by monitoring RU over time yields the binding kinetics for two interacting molecules. (**B**) Enzyme-linked immunosorbent assay (ELISA). ELISA, which is based on the antigen-antibody interaction, effectively profiles the binding of S or its fragment (such as RBD) to an antibody or hACE2. The quantification of binding is evaluated based on the HRP chemiluminescence (conjugated to secondary antibody) or the conjugated fluorescent protein. (**C**) Bio-layer interferometry (BLI) analysis. BLI is an optical technique for exploring the interaction between S and an antibody or hACE2. One of the interacting molecules is immobilized in the streptavidin layer attached to the biosensor, and another interacting molecule remains in the solution. Binding events of each molecule in the biosensor result in the change in the interference pattern when white light is passed through it. The plot of this change over time yields the binding kinetics of two molecules.

The emergence of new SARS-CoV-2 variants and their dissemination into different parts of the world suggest the increased transmissibility and immune escape of the newly emerged variants compared to the early variants. Various approaches, especially in vitro experimental ones, have been rapidly developed and deployed to reveal whether the newly emerged variants have an increased tendency to transmit and evade immune responses [53,63,64,65,66,67,68,69,70,71,72,73,74]. A summary of those analytical tools is informative; however, it is lacking in the literature. Here we provide schematic illustrations to present a simplified overview of these tools, including virus-based (Figure S2) and protein (S)-based approaches (Figure 2). The increased transmissibility can be due to increased viral infectivity, which depends on the S protein. The most commonly used system for SARS-CoV-2 S-based infectivity is the lentivirus (HIV-1), murine leukemia virus (MLV) or vesicular stomatitis virus (VSV) pseudo-typing system, in which the S protein is incorporated into the lentivirus or VSV backbones, and the fluorescent protein or luciferase is used as a signal reporter [53,75,76] (Figure S2A,B). The transmissibility can also be due to the increased fusion capacity of S-carrying viruses or cells into hACE2-expressing host cells. Hence, it can be analyzed by employing virus-to-cell fusion and cell-to-cell fusion assays [53,76,77] (Figure S2C,D). The immune evasion is generally evaluated by a neutralization assay, which measures the potency of the S-directing antibodies (plasma from convalescent patients, sera from vaccinated individuals, and epitope-defined monoclonal antibodies) to neutralize the emerging viral variants either by employing authentic SARS-CoV-2 viruses or pseudo-typed viruses (lentivirus, MLV, or VSV-G pseudo-typed) (Figure S2B) [63,66,67,69,70,74,78,79,80,81,82,83,84,85,86,87,88].

The infectivity, neutralization, and fusion assays are further supported by various protein-level ligand-protein binding techniques, such as surface plasma resonance (SPR, Figure 2A) [89], enzyme-linked immunoassay (ELISA, Figure 2B) [89], biolayer interferometry (BLI, Figure 2C) [76,81,89] and flow cytometry [76]. These methods can yield quantifications depicting binding capacity, dissociation rate, and competitive binding between antibodies or hACE2 and S proteins.

SPR is an optical technique in which one of the two interacting partners is immobilized on a gold sensor chip surface, and the other flows through a microfluidic system that encounters the chip surface. The SPR signal comes from changes in the refractive index at the surface of the gold sensor chip and is measured as the resonance angle. Once the flowing molecule in the microfluidic system binds the immobilized molecule on the sensor chip surface, the refractive index increases, recorded as a change in response, and this response signal is quantified in resonance units (RU). Monitoring the difference in the SPR signal (RU) over time produces a sensorgram, a plot of the binding response (RU) versus time which allows different stages of a binding event to be visualized and evaluated (Figure 2A) [90]. ELISA works on binding specific antibodies (or ligands) to the target antigen and generally uses HRP-conjugated or fluorescent protein-conjugated secondary antibodies to quantify antibody-antigen binding. Direct, indirect, sandwiched, or competitive ELISA techniques can be utilized to identify S-hACE2, S-antibody, or in general, S-ligand interactions, or competitive binding assays (Figure 2B) [91]. Like SPR, BLI is also an optical technique used to measure real-time interactions of the molecules (S-ligand) by analyzing interference patterns of white light reflected from the surface of a biosensor tip. In BLI, one interacting molecule is immobilized in the biosensor, and another remains in the solution. The change in the number of molecules bound to the end of the biosensor tip causes a shift in the interference pattern, which is then plotted as a graph (Figure 2C) [92]. At the cellular level, flow cytometry is widely used to analyze the binding of S protein to antibody or hACE2 [76]. Cells are transiently transfected with a plasmid expressing S or variants and a plasmid expressing a fluorescent protein. Then, 24–48 h post-transfection, cells are incubated with either antibody or hACE2 or both. Secondary antibodies with distinguishable fluorescent labels that are specific against antibody or hACE2 are then used for staining and flow cytometric analyses. This analysis yields the interactions between S and hACE2 or antibodies or inhibitors [76,93,94].

The methods mentioned above, including virological and general protein ligand-binding tools (Figure 2 and Figure S2), can be used in combination to provide an overall picture of the changes in the binding efficiency of the SARS-CoV-2 variants with hACE2 or antibodies, indicative of a shift in transmissibility or immune evasion, as discussed in the following sections. In chronological order, we explore recent findings on the susceptibility of VOCs to antibody neutralization and the binding capacity of the S protein with hACE2 (summary in Table 1). The summary is limited to the studies discussed below or literatures that we have noticed, given the countless research articles on this topic.

### 2.2. Early Variants (Alpha, Beta, Gamma)

The *Alpha/B.1.1.7* variant (also known as 20I/501Y.V1 and VOC-202012/01) emerged in September 2020 in Southeastern England. Several studies have analyzed antigenicity and immune evasion of the alpha variant [67,68,70,74,78,79,80,105,106]. The Alpha variant was implied to be slightly less sensitive to plasma from individuals who have recovered from COVID-19 or sera from individuals who have been vaccinated compared to the USA/WA1 virus [74]. Similarly, sera of 40 participants vaccinated with the mRNA-based vaccine BNT162b2 had slightly reduced neutralizing capacity but retained neutralizing titers against the Alpha S variant relative to the Wuhan (D614) strain [67]. Antibodies isolated from individuals after vaccination with the BNT162b2 vaccine exhibited broad neutralizing potency against the D614G variant and reduced but retained potency against the Alpha variant [79]. Similar results were observed for the sera of convalescent individuals and recipients of an mRNA vaccine (mRNA-1273, Moderna) and a protein nanoparticle vaccine (NVX-CoV2373, Novavax) compared to the D614G variant [78].

Many studies have shown the reduced potency of neutralizing antibodies against the Alpha variant [67,68,70,74,78,79,80,105,106]. NTD-targeting monoclonal antibodies (mAbs) were less effective; meanwhile, a few RBD-targeting mAbs were relatively effective against the Alpha variant compared to the D614 strain [74]. While neutralization potency of some Spike-specific mAbs was severely reduced, polyclonal antibodies isolated from individuals infected in early 2020 were shown to remain effective against the Alpha variant but with less potency compared to the D614G strain [106]. NTD and RBM but not RBD outside of RBM mAbs exhibited reduced potency [79]. Consistent results were obtained from mAbs isolated from SARS-CoV-2-infected individuals, in which 45% of >100 RBD, NTD, and S2 mAbs showed neutralizing activity against the Alpha variant [80]. Mutations in the Alpha S were suggested to confer neutralization resistance to NTD-specific antibodies [80], and neutralization by many mAbs was compromised through light-chain contacts with residue 501 [70].

**Figure 3:**
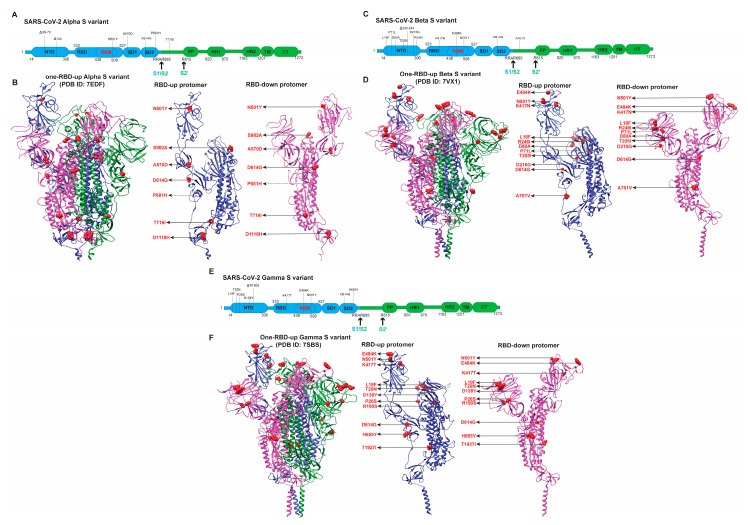
Accumulated mutations in the S early VOCs. (**A**,**B**) Amino acid substitutions are shown in the S domain organization (**A**) and depicted in the one-RBD-up structure (**B**) of the S Alpha variant (PDB ID 7EDF). Amino acid substitutions are highlighted in two different RBD-oriented protomers (RBD-up and RBD-down). The dotted lines indicate the mutated amino acid residues which are not resolved in the structure. (**C**,**D**) Illustrations as in (**A**) and (**B**), respectively, for the S Beta variant (PDB ID 7VX1). (**E**,**F**) Illustrations as in (**A**/**C**) and (**B**/**D**), respectively, for the S Gamma variant (PDB ID 7SBS).

The mutations introduced in the Alpha S (Figure 3A,B) might also affect the binding with hACE2, hence altering virus entry and infectivity. Biolayer interferometry (BLI) analysis showed that the binding of hACE2 to the RBD of the Alpha variant was comparable to that of the S D614G RBD. Still, the dissociation rate constant was slower [107]. This could be the reason for the enhanced transmissibility of the Alpha variant in comparison to S D614G. Another BLI study supported this observation, which showed a significant increase in binding affinity due to the slow dissociation rate [70,71]. Surface plasmon resonance (SPR) and enzyme-linked immunosorbent assay (ELISA) also displayed increased binding of the Alpha S variant to hACE2 [89]. A study also demonstrated a natural selection of N501Y when evolving the RBD to enhance the receptor-binding affinity [108]. Most of the published results imply that the amino acid at 501 is likely a determinant for enhanced hACE2 binding. The results of the Alpha S variant showing increased affinity towards hACE2 support the fast virus spread/transmission, which can be further enhanced by the reduced neutralizing potency of several monoclonal antibodies and sera of vaccinated individuals and convalescent patients.

The *Beta/B.1.351* variant was first identified in South Africa in October 2020. It contains multiple mutations in the S protein (Figure 3C,D), counteracting the S-related host immune response. Analysis of the neutralization capacity of sera of vaccinated individuals or convalescent patients reflects the immune evasion imposed by the Beta S variant. Sera from a group of vaccinated individuals with either the Pfizer-BioNTech vaccine BNT162b2 or the Oxford-AstraZeneca AZD1222 showed a reduction in neutralizing the Beta variant compared to an early Wuhan-related strain [81]. Several RBD-directed mAbs exhibited >10-fold reduction in neutralization titers, or even a complete abolishment. The NTD-directed antibodies lost their neutralization capacity against the Beta variant [81]. Similarly, the decrease in the neutralization capacity of plasma of convalescent patients, plasma of vaccinated individuals, and several monoclonal antibodies that target various antigenic sites within the S protein were extensively reported [43,66,74,96,109,110].

The binding of the Beta S variant to mAbs or hACE2 provides another angle to the shifted fitness [43,81,89]. The BLI analysis demonstrated the profound decrease in the affinity of the Beta S with the mAbs tested [81] and complete abolishment of affinity with antibodies such as two RBD-directed antibodies (G32B6 and C12A2) and two NTD antibodies (C12C9 and C83B6) [43]. The BLI data were also consistent with the binding results to the membrane-bound S trimers measured by the flow cytometry [43]. SPR and ELISA captured the reduced affinity of Beta S with most of the mAbs and abolished affinity with NTD-supersite antibodies [89]. Regarding the binding to hACE2, the affinity of the Beta RBD reported by BLI analysis was 19-fold higher than for the D614 RBD and 2.7-fold higher than for Alpha [81]. This result agrees with that observed by SPR and ELISA [89]. BLI analysis of the Beta S trimer showed a higher affinity for monomeric hACE2 but a slightly lower affinity for dimeric hACE2 than the S G614 trimer [71]. The affinity for hACE2 of the Beta S variant was lower than that of the Alpha S variant [71]. The reduced binding to hACE2 of S beta compared to Alpha remains arguable in the field, although researchers agree upon the enhanced immune evasive ability of Beta over Alpha. In summary, the ability of the Beta variant to evade the human immune response likely outweighed its need for transmissibility after a sizeable human population was infected and interventions had been implemented.

The *Gamma/P.1* variant began to spread from Brazil in January 2021 and had 17 amino acid substitutions, among which 10 were in its S protein (Figure 3E,F). Various studies have been conducted to analyze the immune evasion by Gamma and found that this variant could significantly evade the immune response and challenge the existing vaccines [89,97]. For instance, the plasma from 19 COVID-19-convalescent blood-donors infected months before the emergence of the Gamma variant had 6-fold less neutralizing capacity against the Gamma than the B-lineage isolate SARS-CoV-2/SP02.2020 [82]. Similarly, the plasma collected from individuals after five months of vaccination with a booster dose of the CoronaVac vaccine failed to efficiently neutralize the Gamma variant [68]. Gamma variants exhibited increasing resistance to the majority of a list of neutralizing mAbs, and only a few antibodies remained effective [97]. SPR and ELISA analyses showed that the binding of NTD-directed mAbs DH1050.1 and DH1050.2 to the triple mutant S and S-GSAS-P.1 (or “P.1-like Spike”) remained unchanged, which was in agreement with their retained neutralizing ability against Gamma [89]. BLI analysis showed that the binding of the Gamma variant to hACE2 was profoundly more substantial than that of the G614, possibly due to mutations (K417T, E484K, and N501Y) in RBD [76]. In contrast, the binding of the Gamma S to two RBD antibodies and one NTD antibody was abolished. However, the binding affinity was reduced or unchanged as G614 for a list of other mAbs [76]. Affinities for these antibodies correlated well with their neutralizing abilities [76]. Interestingly, the Gamma variant fused with hosts to a similar extent as G614, Alpha, Beta, Delta, and Kappa at longer time points [76]. RBD mutations of the Gamma variant enhanced receptor recognition, but the same mutations had a more negligible effect on the Delta variant [76]. The dominance of Gamma did not last long, as the Delta variant quickly emerged.

### 2.3. Delta/B.1.617.2 Variant

**Figure 4:**
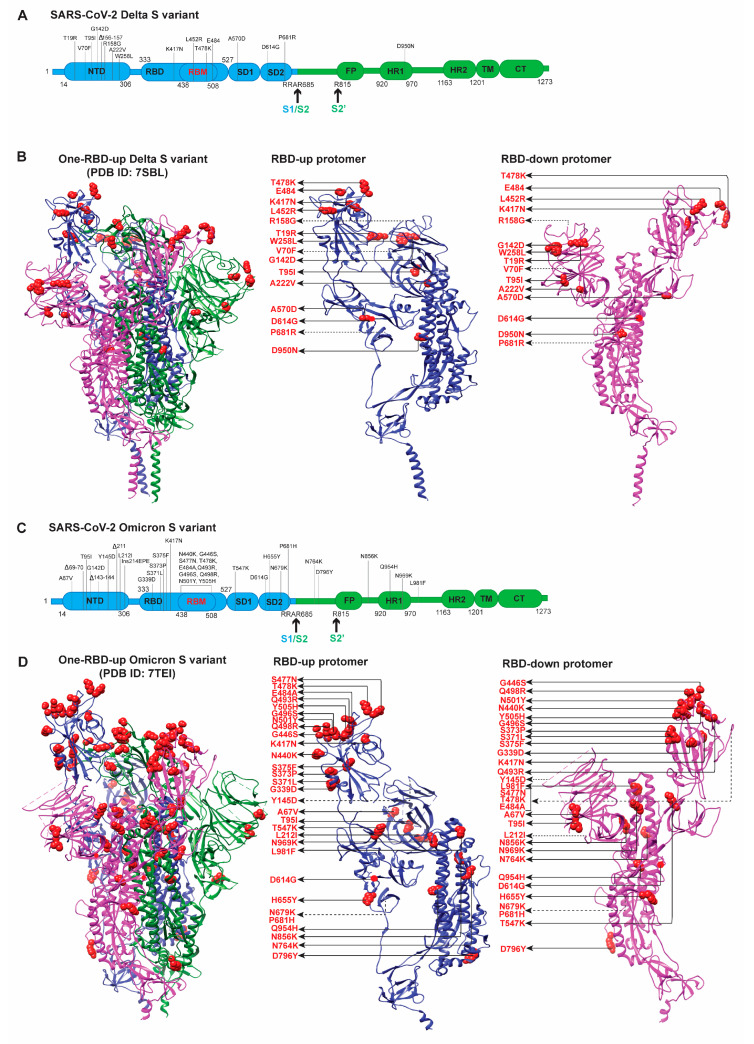
Accumulated mutations in the S Delta and Omicron variants. (**A**,**B**) Amino acid substitutions are shown in the S domains (**A**) and depicted in the one-RBD-up structure (**B**) of the S Delta variant (PDB ID 7SBL). Mutated amino acid residues are highlighted in the RBD-up and RBD-down protomers. The dotted lines indicate the mutated amino acid residues which are not resolved in the structure. (**C**,**D**) Illustrations as in (**A**,**B**), respectively, for the S Omicron variant (PDB ID 7TEI).

The *Delta/B.1.617.2* variant, initially widely spread in India in March 2021, soon outcompeted other variants and became the dominant worldwide pandemic form. The increased transmissibility and escape from the immune responses due to the accumulation of mutations, especially in the S protein (Figure 4A,B), were believed to account for the rapid spread of the Delta variant across the globe [66,69,76,83]. The antigenicity, transmissibility, and immune evasion were determined by analysis of neutralization of the virus by sera from vaccinated individuals, convalescent patients, and neutralizing mAbs, binding affinity with the mAbs and hACE2 [66,69,76,83].

Various studies of the Delta variant were performed as it became the rapidly spreading SARS-CoV-2 variant. Serum neutralizing titers against the Delta variant were lower in ChAdOx1 vaccinated individuals than in BNT162b2 vaccinated individuals [66,83], and neutralization of the Delta variant by sera from both vaccinated groups was profoundly lower than the Wuhan-Hu-1 [83]. Delta Spike-carrying pseudo-typed viruses exhibited compromised sensitivity to monoclonal antibodies targeting the RBD and the NTD [83]. Compared to the D614G, the same trend of increased resistance of the Delta variant to convalescent sera and vaccine-induced antibodies was also observed [83]. In addition, the Delta variant is more resistant to neutralization than the Alpha variant: (1) sera collected from convalescent individuals up to 12 months after the onset of symptoms were 4-fold less potent in neutralizing against another variant from the same lineage, (2) sera from individuals who had received one dose of the BNT162b2 or the AstraZeneca vaccine had a similar inhibitory effect on the Delta variant, (3) administration of two doses of the vaccine generated a neutralizing response in 95% of individuals, however, the potency was lower against the Delta, and (4) the Delta variant was more resistant to neutralization by a few anti-NTD and anti-RBD monoclonal antibodies, including bamlanivimab [69]. The ligand-binding BLI assay and flow cytometry assay showed that the Delta S only lost binding to the two NTD antibodies with little change in affinities for the other antibodies, including those targeting the RBD [76]. The Delta variant fused to the host cell membrane more efficiently at low levels of hACE2 and infected target cells considerably faster than the other variants (G614, Gamma, Kappa, Alpha) [76]. hACE2 did not dissociate more rapidly from the Delta RBD, which might contribute to its transmissibility [76].

### 2.4. Omicron/B.1.1.529 Variant

*Omicron* is the latest variant which is spreading very fast globally. The Omicron variant evolved by acquiring more mutations than the previously emerged variants (Figure 4C,D). It has more than 30 mutations in its S protein, hence significantly affecting the hACE2 and antibodies binding and antibody neutralization. An increasing number of studies have revealed that Omicron could evade the host immune response [63,65,75,86,87,88,100,111]. Many studies revealed that vaccine boosters were moderately helpful to mitigate the Omicron infection [63,75,84,85,86,87,88,100], but could enhance immunity against Omicron [85,87,100]. For instance, sera from two groups of vaccinated (Pfizer) individuals who were or were not previously infected neutralized the D614G virus more profoundly than the Omicron variant, in which the Omicron variant demonstrated a 22-fold escape from vaccine-elicited neutralization in both groups [84]. A similar reduction was shown in other studies [63,75,86,87,88,100]. However, to a moderate extent, sera from individuals who received vaccines or boosts remain effective against the Omicron variant, although the efficacy is lower [63,84,87,88,100]. Fortunately, vaccine boosters can enhance immunity against the Omicron variant [85,87,100]. For instance, administering a Pfizer booster dose and vaccination of previously infected individuals generated an anti-Omicron neutralizing response but lower than that against the Delta variant [85]. The longer interval between the vaccine doses helps develop a better immune response with a less pronounced reduction in the neutralization of the Omicron variant [88].

From the perspective of immune evasion, Omicron exhibits increased resistance to neutralization by mAbs in a handful of studies [75,85,87,88]. The neutralizing ability against Omicron of 17 mAbs among a panel of 19 mAbs was either abolished or impaired [87]. Similarly, another nine mAbs had very low potency or no effect in neutralizing the Omicron variant [85]. The neutralizing activity of the NTD-directed antibodies 4–8 and 4A8 was abolished for the Omicron variant, likely due to the deletions in the NTD (Δ69–70 and Δ144–145) [88]. Complete abolishment or impairment was observed for RBD-directed antibodies, such as ab1, ab8, S309, and S2M11 [88].

Similarly, the analysis of eight clinical monoclonal antibodies (LY-CoV555, LY-CoV016, 175 REGN10933, REGN10933, CoV2-2130, CoV2-2196 and CT-P59, and Sotrovimab) often used as two-mAb cocktails during treatment showed reduced neutralizing abilities against Omicron [63,111]. Seven of these mAbs bind to epitopes overlapping with the RBM, thus blocking the binding of S to hACE2 [111]. Only Sotrovimab does not block hACE2 binding and it neutralizes SARS-CoV-2 by targeting non-RBM epitopes [111,112]. Sotrovimab showed 3-fold reduced potency against Omicron and Omicron-R346K variants compared to the Wuhan strain [63]. In contrast, the combination of CoV2-2130 and CoV2-2196 showed about a 100-fold reduction in neutralizing activity against the authentic Omicron SARS-CoV-2 compared to the Wuhan strain [63]. Meanwhile, only 1 of 5 NTD, 3 of 22 RBM, and 1 of 9 RBD mAbs retained neutralization potency against Omicron but with reduced potency [63]. Fortunately, mAbs that target a highly conserved cryptic site retained neutralization against Omicron, emphasizing the importance of targeting conserved epitopes for broad neutralization breadth and effectiveness in the case of antigenic shifts [63]. This conclusion is further supported by the retained neutralizing activity of about 15% of the tested antibodies against Omicron [65]. RBM-targeting neutralizing mAbs lost their neutralization capacity against Omicron while neutralizing antibodies that target conserved epitopes such as the S309 and CR3022 sites essentially retained neutralization potency [65]. Multiple synergistic mutations on the S epitopes were more effective in escaping from neutralizing mAbs than the single mutation [65].

The Omicron studies we discussed above and numerous other not discussed ones reached the same conclusion of the superior immune evasive and transmissible abilities of Omicron compared to previous VOCs. However, the hospitalization rate, hospital attendance and death caused by Omicron are lower than Delta [113,114]. The lower hospitalization rate is likely due to many factors, such as increased vaccinations and boosters in the human population and the implied lower pathogenicity of Omicron than Delta in an increasing number of in vitro and in vivo studies [115,116,117].

## 3. Structural Plasticity/Flexibility

As discussed above, the S protein is required for the virus attachment with the host cell in the viral entry process, interaction with existing neutralizing antibodies, and triggering host adaptive immune response. Mutations acquired in S during virus evolution can potentially affect the local structures and/or overall conformations of the S protein, thus affecting its biological functions. Any modifications on S could affect the structure and alter the binding affinity to hACE2 and antibodies. The mutations in S protein could also change the electrical surface potential on S, which impacts the interaction with hACE2 [118]. Hence, the structural surveillance of S variants is significant, which can offer molecular interpretations of altered virus transmissibility and immune responses and lay the groundwork for developing novel S-based anti-SARS-CoV-2 interventions. We will review the high-resolution structures of S variants and associate the structural changes imposed by mutations with the function of S. We will start with the early VOCs, including the Alpha, Beta, and Gamma S variants.

### 3.1. Early Variants (Alpha, Beta, Gamma)

*Alpha/B.1.1.7* has seven mutations and three deleted residues referring to the Wuhan strain S (Figure 1 and Figure 3A,B), including three deleted residues within NTD, one modification (N501Y) within RBM, three in the CTD, and three within S2. A cryo-EM study reported a 1.8:1 RBD-up/RBD-down conformation of Alpha S, more than the 0.8:1 RBD-up/RBD-down of D614G S [89]. Another cryo-EM analysis of Alpha S protein identified four conformations, including three slightly different one-RBD-up and one two-RBDs-up conformations [107]. In situ parallel smFRET and cryoET analyses of Alpha S in the context of the same lentivirus revealed four virus-associated conformations: three-RBDs-down, one-RBD-up, two-RBDs-up, and three-RBDs-up conformations [73]. Alpha S tends to occupy more one-RBD-up than two-RBDs-up [73,89,107]. The missing three-RBDs-down in the cryo-EM structure is likely due to the increased RBD mobility, which lowers the threshold to transition from three-RBDs-down state to RBD-up states, in addition to the classification approach during cryo-EM data analysis. It is also possible that mutations in Alpha S might weaken contacts between RBD and adjacent NTDs in the three-RBDs-down state [89] and enable the increased presentation of receptor-accessible RBD-up states. In the Alpha S, mutations lie in multiple domains that are present away from the RBD/NTD region, such as A570D (SD1), P681H (SD2), S982A (HR1), D1118H (CD), and T716I (linker between SD2 and fusion peptide). The P681H site near the furin cleavage site was not resolved by cryo-EM due to its disordered form [89,107]. Notably, the accumulation of stabilizing contacts due to amino acid substitutions was observed [75,87], which might stabilize the pre-fusion S to prevent premature S1 shedding in Alphas S [107]. The aromatic side chain of mutated Y501 of Alpha S interacts with hACE2 by binding with K353 and Y41, facilitating the formation of the S–hACE2 complex [118]. This complex formation is further stabilized by a newly formed π–π stacking between the Y501 of Alpha S and Y41 of hACE2 [118]. A contracted rigid-body domain movement of hACE2 towards the RBD of Alpha could explain the increased affinity of Alpha S with the hACE2 receptor [107]. Thus, the synergic effect of a more stable Alpha S structure, stabilized contacts between S and hACE2, the deletion of three residues in NTD, and N501Y are most likely responsible for the higher transmissibility and slightly enhanced immune escape of Alpha (a summary in Table 1) than the previous D614G variant.

For *Beta/B.1.351*, cryo-EM studies of the Beta S variant have shown that Beta S tends to occupy more open conformational states [71,109,119,120]. The all-RBDs-down and one-RBD-up (Figure 3C,D) structures of Beta S are almost identical to those of G614, in which the three critical mutations in RBD: N501Y, K417N, and E484K (Figure 3C,D), do not cause significant structural rearrangements [71]. Two cryo-EM structures differentiated the tilting of RBDs in two one-RBD-up configurations of the Beta S trimers and thus revealed a transition state [120]. The tilting angle of RBD in a protomer was lower in one structure than in the other and was thus categorized as a transition state. This transition state has a similar conformation as a one-RBD-up state except for the missing density for large parts of RBM and the dynamic nature of RBD. Interestingly, Beta S trimers exhibited almost equal percentages in these two states [120]. The RBDs of the Beta S trimer generally appeared to be less compact because of greater twist angles in NTD compared to the G614 open–state Spike and slight movement of RBDs [120]. Several structural configurations of the Beta S trimer complexed with hACE2 have been resolved by cryo-EM, including S-hACE2-C1 (only RBD-1 up), S-hACE2-C2a (RBD-1 and RBD-2 up), S-hACE2-C2b (RBD-1 and RBD-3 up), and S-hACE2-C3 (all three RBDs up) [120]. The resolved models showed that hACE2 binding to Beta S trimer induced a pronounced S-trimer population shift to the more open C2a/C2b or fully open C3 states, promoting the shedding of S1 and succeeding post-fusion state. The interaction of RBD-1 with hACE2 induced an upward tilt of the RBD-1 [120]. Due to the mutation in K417N in Beta S, the original salt bridge formed between RBM K417 and hACE2 D30 was found to be lost in cryo-EM structures; however, the loss was compensated by the N501Y substitution that formed a new hydrogen bond between Y501 and K353 on hACE2 [71,120]. The N501Y substitution also established more aromatic interactions with Y505 of RBD and the Y41 of hACE2 [120]. These interactions mediated by N501Y could explain the higher hACE2-binding affinity of the Beta S trimer relative to those of the D614 and G614 S. Similarly, cryo-EM structures of Beta S revealed a highly populated, fully open state, i.e., all three RBDs in the up conformation (three-RBDs-up) in addition to the partially open states (one/two-RBDs-up) [109]. The electrostatic distribution on the RBD was modified by the mutations K417N and E484K, causing the loss of salt bridges between Lys417 and hACE2 Asp30 and Glu484 and hACE2 Lys31 [71,109]. Detailed structural information of Beta S complexed with eight neutralizing antibodies revealed that five of them directly antagonized the binding of hACE2, while the other three were independent upon the hACE2 binding [119]. This result agrees with the increased immune escape of the Beta variant [81,96,97]. Altogether, Beta S samples more open conformations, thus increasing the chance of binding to hACE2. K417N and E484K likely cause the reduction of neutralization by RBD-directed antibodies. The loss of two salt bridges between RBD and hACE2 is compensated by the gain of hACE2-binding by N501Y. Besides the three critical mutations in the RBD, more mutations in NTD largely change the NTD antigenic surface and enhance the resistance to neutralization by NTD-directed antibodies. Overall, compared to the Alpha variant, the emergence of Beta variants likely favor immune evasion more than virus transmissibility.

For *Gamma/P.1*, the overall structure of P.1/Gamma Spike protein is similar to that of the parental G614 S trimer in the respective conformation [55,76]. We used the one-RBD-up structure of the Gamma S trimer to demonstrate the amino acid substitutions and relative movements between RBD-down and RBD-up (Figure 3E,F). The cryo-EM analysis of the full-length Gamma S trimer showed two classes of one-RBD-up conformations [76], and a truncated version only showed one primary class of one-RBD-up [97]. Likely, the all-RBDs-down closed structure of Gamma S was not captured by cryo-EM. In one-RBD-up Gamma S, 1 FPPR (fusion peptide proximal region, residues 828 to 853) and 630 loops (residues 620 to 640) were proposed as control elements for stabilizing S trimer [76]. Besides, the effects of different mutations on the Gamma S were detailed in atomic structures and rationally interpretated [76]: (1) mutations located near NTD regions (L18F, T20N, P26S, D138Y) and R190S modified the structure in the NTD region of Gamma S and participated in restructuring a new conformation which stabilizes the 70 to 76 loops, (2) T20N faciliated the formation of a new glycosylation site, and (3) three RBD mutations (K417T, E484K, and N501Y) in Gamma S work in a cooperative manner to maintain the structural intergrity of S for hACE2 binding. Like in Alpha S, 501Y of Gamma S forms a complex with hACE2 through 353K and 41Y. However, this increase in hACE2 affinity was nullified by the other mutation, K417T. The negative charge of E484 residue could form an electrostatic bridge with the cationic charge of K31 of hACE2. However, the formation of the bridge can be abrupted by E484K which increases the charge repulsion of K484 and K31 of hACE2, (4) H655Y in the CTD2 did not change the local structure but might play a role in destabilizing the trimeric structure due to its proximity to the N terminus, (5) the T1027I mutation in the S2 region had no effect on the S structure, and V1176F was not resolved. Numerous computational and modeling studies of S variants have provided additional insights into how the altered electrostatic networks were imposed by key mutations on S–hACE2 interactions [118,121,122,123,124]. For instance, an insilico analysis of the effect of K417T, E484K and N501Y on the electrostatic surface potential of the RBD showed an increase in the positive potential of the central area of RBD, and thus warranted affinity for negatively charged lipid-raft gangliosides which were thought to interact with the NTD domain of S [118]. This insilico study also showed that the side chain of K484 formed stabilizing interactions with the methylene groups of K31 (van der Waals) and Y83 (hydrogen bond) of hACE2, whereas the K417T mutation can abrogate the contact between this residue and the hACE2 [118].

Similar to the case of Beta S, K417T and E484K substitutions might also impair the binding and neutralization of the Gamma variant by RBD antibodies [76]. E484K mutation was shown to interrupt its sidechains interaction with the Phe490 backbone through a hydrogen bond, resulting in the reduced binding of class 2 RBD-directed neutralizing Abs such as DH1041 and DH1043 [89]. The rearrangement of antigenic sites in the NTD could result in the loss of activity of many NTD-directed antibodies [76,97]. The effect of D138Y substitution in the center of the NTD supersite could impact NTD-directed antibodies such as 2–17 and 4–19 [97]. Cryo-EM analyses also indicated that the E484K mutation weakened RBD-to-RBD coupling and increased the RBD-up Spike populations [89]. Another cryo-EM study showed one-RBD-up conformation as the most favorable for Gamma S [97]. Beta and Gamma variants share very similar immune evasive profiles, likely due to the shared critical mutations in S (Figure 3C–F).

### 3.2. Delta/B.1.617.2 Variant

The overall architectures of full-length Delta S were similar to those of G614, Beta, and Gamma S trimers in the corresponding states [76]. Delta S does not contain the two critical RBD mutations (E484K and N501Y) existing in Beta and Gamma variants (Figure 4A,B). Cryo-EM analysis of Delta S trimer has resolved three structures: one all-RBDs-down and two one-RBD-up conformations [76]. Like in the G614 S trimer, all the FPPR and 630-loop pairs were structured in the all-RBDs-down conformation, whereas only 1 FPPR and 630-loop pair was ordered in the one-RBD-up conformation [76]. An N-linked glycan at Asn343 had a substantial density that might have a role in RBD opening. The distal end of this glycan interacted with the adjacent RBD, forming a ring-like density, and stabilizing the three-RBDs-down conformation [76].

Delta S contains three point mutations (T19R, G142D, and E156G) in the NTD domain and a two-residue deletion (F157del and R158del) (Figure 4A,B). These altered features in NTD appear to restructure various parts of the Spike protein: (1) reconstruction of the 143–154 loop, which has an N-linked glycan (N149), helps a part of the NTD epitopes projecting away from the viral membrane, and (2) reconstruction of the N-terminal segment and the 173–187 loop leads to the alteration of the antigenic surface near the NTD epitopes, the primary reason for the abolishment of binding and neutralization by NTD antibodies [76]. D950N substitution in HR1 (heptad repeat 1) of S2 does not appear to affect the S structure [76]. Mutations L452R and T478K do not lie on the hACE2 contacting surface. The combination of these two mutations exerts no significant effect on the S structures, a slight impact on the hACE2-binding [125], and no effect on the binding/neutralization of many RBD-directed neutralizing antibodies [39,76]. Interestingly, RBD mutations accumulated in Delta S, although they reside in the most immunogenic site, have a minimal impact on the hACE2 binding [125]. Nevertheless, T478K substitution and the increased propensity for adopting more RBD-up conformations were suggested to play a critical role in enhancing the binding affinity of Delta S to hACE2 [99]. Delta S-dependent lentivirus fused to the host cells more rapidly than Gamma and Kappa, likely contributing to the fast spread of the Delta variant [76]. There is still no consensus on whether Delta S has a higher binding affinity to hACE2 [76,99,125], given that the N501Y does not exist in Delta S (Figure 4A,B). There is considerable agreement on the antigenic shifts in the NTD and the antigenic preservation in the RBD [76,99,125]. The remodeling of the NTD supersite is largely responsible for the immune evasion of the Delta variant [76,99,125]. However, there is no clear answer as to why some RBD antibodies fail to effectively neutralize the Delta variant (especially without the E484K mutation) [69].

### 3.3. Omicron/B.1.1.529 Variant

The Omicron S variant contains the most mutations in the Spike protein, with 15 in the RBD responsible for binding to hACE2 for virus entry (Figure 4C,D) and several deletions/mutations in the NTD. The mutations acquired by the Omicron S protein are dispersed both on the surface and the interior of the Spike protein. The RBD modifications primarily present on one face of the domain, distributed in hACE2 and several neutralizing antibodies binding interfaces (Figure 4C,D). With this large number of mutations in the two antigenic “supersites”, RBD and NTD, it is expected that Omicron is more immune-evasive than its ancestors. Indeed, the extensive modifications in S facilitate Omicron escape from being neutralized by antibodies (mAbs, sera, or plasma) elicited by pre-infection or vaccination [65,84,85,87,101,102]. RBD binds to hACE2 for virus entry; however, 15 mutations in the RBD do not appear to cause the reduction in its ability to bind hACE2 [101,102,103,126]. The atomic details of Omicron S ectodomain structures provide insights into how Omicron escapes antibodies and retains its affinity to bind hACE2 for virus entry [102,103,104,126,127].

The overall ligand-free, hACE2-complexed, or mAb-complexed Omicron S structures [102,103,126] are very similar to other S variants [23,27,57,128], although differences are found in the RBD packing [127] (Figure 4D) as well as in NTD and RBD antigenic surfaces remodeling [104]. The fusion peptide in the Omicron S variant is more flexible, and RBD is more tightly packed than other variants (Figure 4D) [127]. The tightly packed RBDs make RBD-up and RBD-down protomers in one-RBD-up trimeric conformation look differently than other S variants (Figure 4), which is not seen in other reported Omicron structures [101,102,103,104,126]. Most structural studies of the Omicron S ectodomain only revealed either all-RBD-down or one-RBD-up or both conformations [101,102,103,104,126,127]. The revealed architectures are not as informative as the local structural changes in RBD and NTD with/without hACE2 contacts [101,102,103,104,126,127].

The Omicron variant has triple mutations (K417N, T478K, and N501Y) in RBD, similar to previous VOCs, and their effect on the ACE2 binding has been extensively studied [88,89,128]. The N501Y was reported to enhance hACE2 binding, whereas K417N was considered as an affinity-reducing mutation [88,89,128]. E484A, not E484K, in Omicron S, might cause loss of polar interactions between RBD and hACE2. Several other mutations (S477N, Q493R, G396S, Q498R, and N501Y) are present in the S–hACE2 interface. Structures of hACE2-complexed structures showed that newly formed hydrogen bonds and salt bridges between these residues (S477N, Q493R, G396S, Q498R, and N501Y) and hACE2 residues could compensate for the loss of interaction with hACE2 caused by K417N and E484A [101,103,126]. Therefore, diverse mutations in the Omicron S protein contribute collaboratively to the retention of hACE2-binding affinity and the increase of antibody evasion, explaining its high transmissibility and predominance over the Delta variant.

## 4. Conclusions

Since the beginning of the COVID-19 pandemic, SARS-CoV-2 VOCs that are more transmissible and possibly more pathogenic have emerged. The currently administrated vaccines and antibody therapy are primarily directed against the SARS-CoV-2 S protein as the target of the antibody response [33,34,35,36,37,38]. As a large population has contracted SARS-CoV-2, the virus has been under selection pressure to evolve, adapt to the human host, and evade immune responses, primarily by mutating the S protein. On the bright side, the rapid vaccine rollout and monoclonal antibody therapies have been implemented, effectively reducing the risks of SARS-CoV-2 infection-associated severity and death. On the other hand, the emergence of new variants may continue to dampen immune recognition, threaten to prolong the pandemic, and likely necessitate vaccine booster shots with S immunogens covering new variants. This review discussed the S-related immune evasion of once-dominant circulating SARS-CoV-2 VOCs worldwide. Based on the available structures of S VOCs, we aimed to provide an overview of the effects of accumulated mutations on the overall conformational equilibrium and local structures of S, which govern the alternations in its antibody recognition and receptor engagement. The understanding of the correlation between structural changes in S and the altered virus transmissibility and immune evasion of SARS-CoV-2 VOCs during virus evolution informs the structure-aided design of S-centric vaccine boosters and therapeutics to curtail the COVID-19 pandemic.

## Figures and Tables

**Table 1:** Susceptibility of SARS-CoV-2 VOCs to neutralization. The overall trends and changes of antibody neutralization capacity against different variants and binding capacity of RBD with hACE2 are listed in the table. The referred S strain is enclosed inside parentheses. The extent of reduction or increase varies significantly across different literatures, due to the differences of characterization assays (virus-based, cell-based, and protein-based) and the variability of samples (sera, plasma, virus, antibodies, S ectodomain, or S RBD). The results are limited to literatures discussed in this review. We are aware of the discrepancy and even inconsistency in different research articles.

SARS-CoV-2 Variants	Neutralizing Capacity				Binding Capacity of RBD with hACE2
	Plasma from Convalescent Patient	Sera from Vaccinated Individuals	RBD-Directed Antibodies	NTD-Directed Antibodies	
**Alpha**	Slightly reduced: >1-fold (compared to D614G) [70,74,78,95]	Reduced: >2-fold (D614G) [67,70,74,78,95]	Reduced: wide range >1- to >100-fold to total loss [70,74,79,95]	Completely reduced: total loss of neutralization [74,79,80]	Comparable and noticeably increased: >2-fold (D614G) [70,89]
**Beta**	Reduced: >7-fold (D614G) [81,95,96]	Reduced: >7-fold (D614G) [81,95,96]	Reduced: wide range >1- to >100-fold to total loss (D614G) [81,95,96]	Completely reduced: Total loss of neutralization [96]	Increased: >15-fold (Victoria Strain) [81]
**Gamma**	Reduced: >3-fold (B lineage isolate) [82,95,96,97].	Reduced: >3-fold (D614G) [82,96,97].	Reduced: wide range >1- to >100-fold reduction to total loss (D614G) [95,96,97].	Dramatically or completely reduced [82,96,97]	Increased: >18-fold (Victoria strain) [76,98]
**Delta**	Reduced: >5-fold (D614G) [83,95].	Reduced: >5 to >10-fold (D614G WT), 3- to 5-fold (Alpha) [66,69,83].	Reduced: wide range 2- to >40-fold to total abrogation (D614G) [69,83,95].	Dramatically or completely reduced [69,83]	Increased: >10-fold (D614G) [76,99]
**Omicron**	Dramatically reduced: >70-fold to total loss (D614G) [63,75,84,85,86,87,100].	Dramatically reduced: >35- to <60-fold (D614G) [63,75,84,85,86,100].	Reduced: wide range >1- to >100-fold to total loss (D614G) [63,85,87,101]	Completely reduced: [63,101]	Increased: <10-fold (D614G) [63,102,103,104].

## Data Availability

Not applicable.

## Supplementary Materials

The following supporting information can be downloaded at: https://www.mdpi.com/article/10.3390/v14061255/s1, Figure S1: Superimposition of uncleaved (PDB 6ZGE) and furin-cleaved (PDB 6ZGI) three-RBDs-down structures of SARS-CoV-2 S trimer; Figure S2: Schematic demonstration of various virological techniques applied for analyzing SARS-CoV-2 immune evasion and transmissibility.
