# Supplementary material for: Structural Plasticity and Immune Evasion of SARS-CoV-2 Spike Variants
Source: Viruses. 2022 Jun 9;14(6):1255. doi: 10.3390/v14061255 (PMC9229035; doi:10.3390/v14061255)
Supplement: Supplementary file 1 [file viruses-14-01255-s001.zip › viruses-1710447-supplementary.pdf]

viruses

Supplementary Materials:

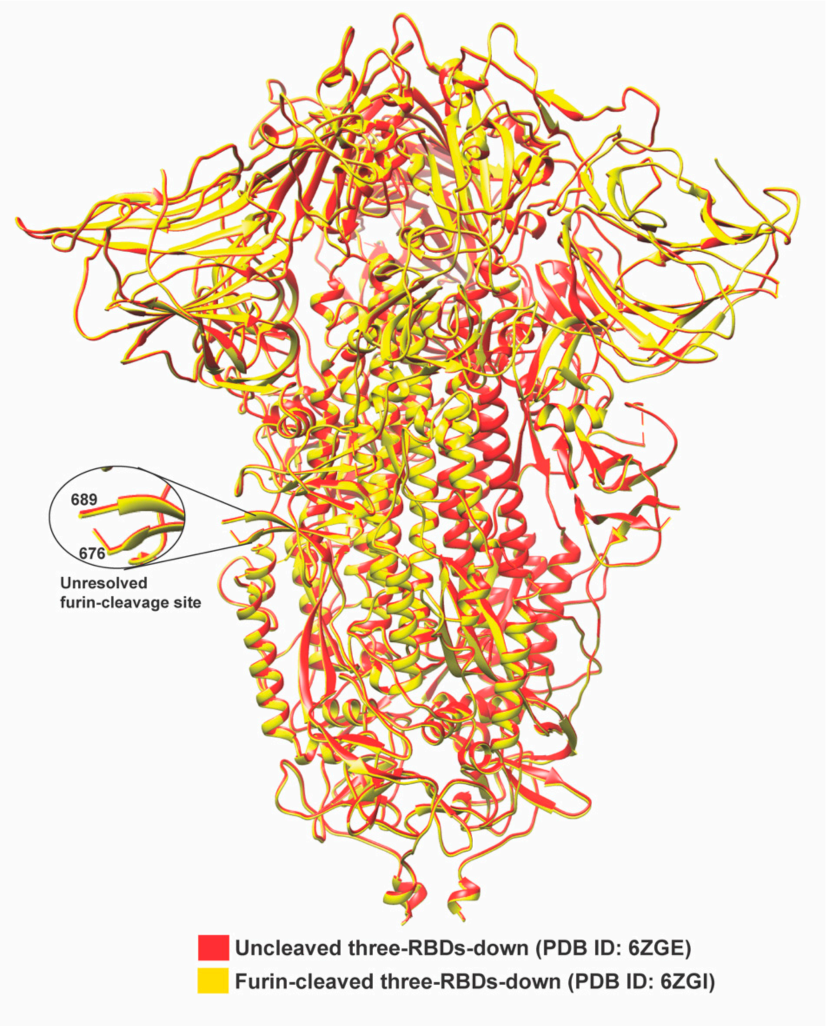

Figure S1. Superimposition of uncleaved (PDB 6ZGE) [51] and furin-cleaved (PDB 6ZGI) [51] three-RBDs-down structures of SARS-CoV-2 S trimer. Uncleaved S structure is shown in red, and the furin-cleaved is in yellow. The furin cleavage site (682-RRAR-685) at S1/S2 is not resolved in both structures.

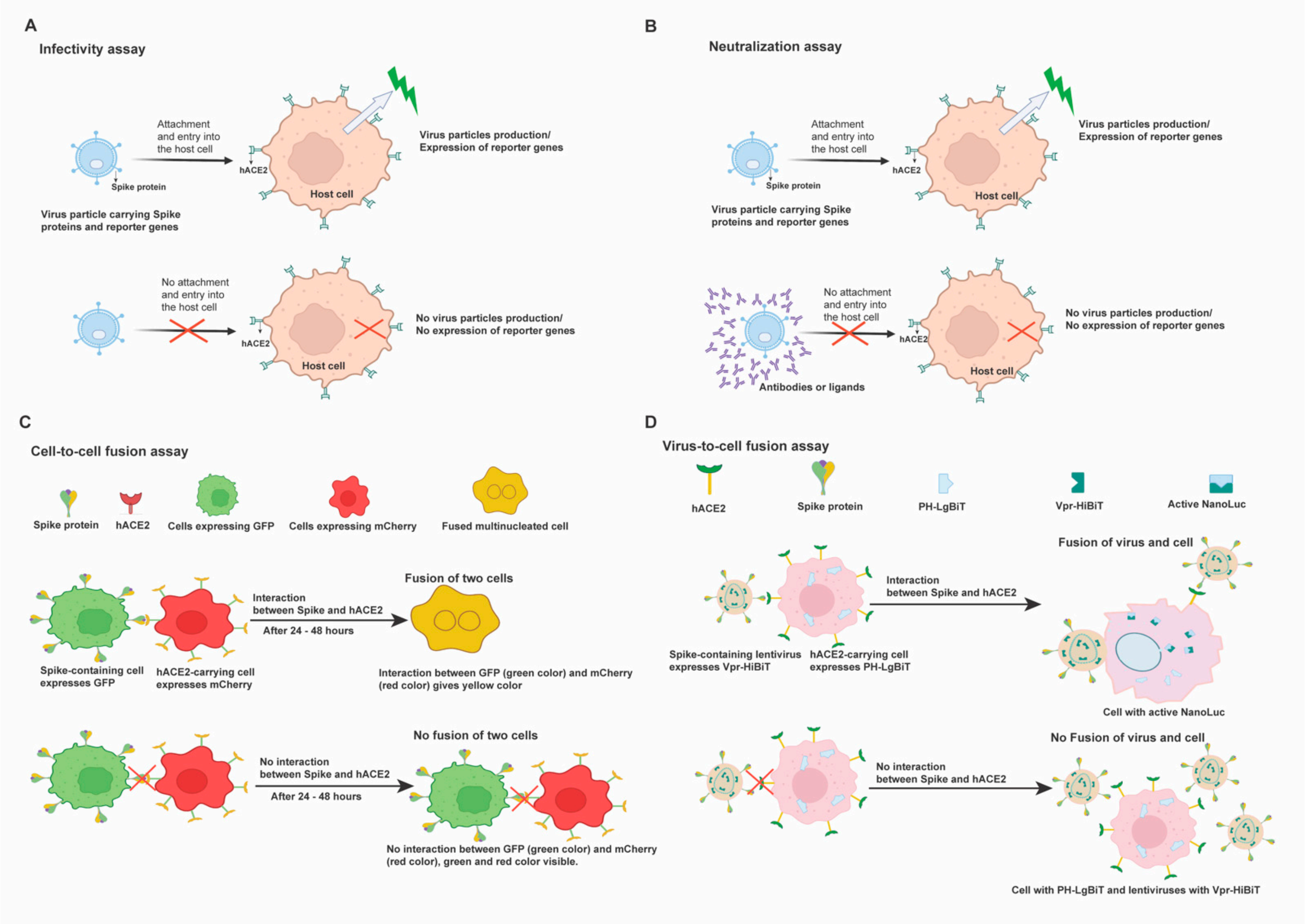

Figure S2. Schematic demonstration of various virological techniques applied for analyzing SARS-CoV-2 immune evasion and transmissibility. (**A**) Pseudovirus infectivity assay. For the demonstration, lentiviruses decorated with SARS-CoV-2 Spike packaged together with reporter genes like luciferase gene are used to infect target cells expressing hACE2. The expression of reporter genes is then measured and quantified to inform virus infectivity. (**B**) Pseudovirus neutralization assay. Lentiviruses or other types of virus particles decorated with SARS-CoV-2 Spike consisting of reporter genes like luciferase gene are firstly incubated with antibodies (plasma from convalescent patients, sera from vaccinated individuals, or monoclonal antibodies) and used to infect hACE2 expressing target cells. Typically, 24 hours or longer after infection, the expression of reporter genes is measured, and the level of neutralization by antibodies is determined. (**C, D**) The virus-to-cell and cell-to-cell fusion assays exploit the complementation of the split reporter protein system like GFP-mCherry interaction system (**C**) [77], split nanoLuc system (**D**) [53] or α-complementation of *E. coli* β-galactosidase [76]. (**C**) An example of the cell-to-cell fusion assay. Two cells are transiently expressing either the combo of GFP and the Spike protein or the combo of mCherry and hACE2. Both cells are then mixed for some time (24-48 hours) to allow Spike-hACE2 dependent fusion. The fusion of the cells will be in yellow, whereas unfused cells will be either green or red. The light intensity from the reporter protein reflects the extent of fusion events between two cells following the interaction between S and hACE2. (**D**) A virus-to-cell fusion assay based on the HiBit and LgBiT split NanoLuc system. Vpr-HiBit is packaged into S-carrying lentiviral particles. These particles are then used to infect target cells that transiently express LgBiT tagged to the PH domain of human phospholipase Cd at the N terminus alone or together with hACE2. 24-48 hours post-infection, reconstituted NanoLuc activity reflects hACE2-dependent virus-to-cell fusion.
